# Effects of Antioxidative Lactic Acid Bacteria on Bacterial Community Dynamics, Physicochemical Properties, and Oxidative Stability During Dry-Cured Fermented Sausage Processing

**DOI:** 10.3390/foods15193419

**Published:** 2026-09-24

**Authors:** Rong Tang, Hui Wang, Xingying Dai, Biqin Liu, Huihua Tang, Xing Wan, Zheng Zhou, Junfei Chen, Qiao Shi

**Affiliations:** 1 Agro-Product Processing Research Institute, Yunnan Academy of Agricultural Sciences, Kunming 650221, China; 15288408019@163.com (R.T.);; 2 Department of Microbiology, Faculty of Agriculture and Forestry, University of Helsinki, 00790 Helsinki, Finland; 3 State Key Laboratory of Meat Quality Control and Cultured Meat Development, MOST, Key Laboratory of Meat Processing, MARA, Jiangsu Collaborative Innovation Center of Meat Production, Processing and Quality Control, College of Food Science and Technology, Nanjing Agricultural University, Nanjing 210095, China

**Keywords:** *Leuconostoc carnosum*, antioxidant activity, bacterial diversity, lipid oxidation, protein oxidation

## Abstract

This study aimed to screen lactic acid bacteria (LAB) with enhanced antioxidant activity and evaluate their potential as starter cultures for dry-cured fermented sausages by assessing their effects on oxidative stability and physicochemical quality during processing. The antioxidant activities of different cellular fractions of five LAB strains were first compared, and the results indicated that the antioxidant activity was mainly associated with extracellular metabolites. Based on their good in vitro antioxidant activities and oxidative tolerance, *Leuconostoc carnosum* AMS1 and *Lactiplantibacillus plantarum* P4 were subsequently selected as starter cultures for sausage fermentation. Individual inoculation with AMS1 and P4 modulated the bacterial community structure during fermentation and reduced the abundance of opportunistic pathogens. Compared with the uninoculated control, starter inoculation accelerated acidification, reduced water activity, and positively modulated color and texture properties. Moreover, inoculation improved oxidative stability, as indicated by reduced thiobarbituric acid reactive substances (TBARS) levels and lipoxygenase (LOX) activity, and alleviated protein oxidative changes, as evidenced by lower carbonyl accumulation, reduced sulfhydryl group loss, and a lower increase in surface hydrophobicity. In conclusion, AMS1 and P4, particularly AMS1, showed potential as antioxidative starter cultures for dry-cured fermented sausages by improving physicochemical quality and oxidative stability during fermentation and maturation.

## 1. Introduction

Dry-cured fermented sausages are valued for their distinctive organoleptic characteristics and high nutritional quality. During processing and subsequent storage, lipids, proteins, and carbohydrates undergo a series of complex biochemical reactions that collectively determine the quality attributes of the final product [1]. Among these, lipid oxidation is a critical factor influencing the flavor, quality, and shelf life of dry fermented sausages. It promotes the oxidative degradation of unsaturated fatty acids, generating secondary oxidation products such as aldehydes and ketones. Excessive lipid oxidation can cause off-flavors and accelerates product deterioration [2,3]. Similarly, excessive protein oxidation induces modifications of amino acid side chains, protein cross-linking, and structural alterations, leading to reduced solubility, impaired emulsifying capacity, and decreased water-holding capacity, all of which adversely affect sausage texture, flavor release, and digestibility [4]. Given the potential health risks associated with oxidation products and the declining consumer preference for synthetic antioxidants, the development of natural and safe antioxidant alternatives has become a major research priority.

The microbial community of fermented sausages is highly diverse, and its compositional dynamics significantly influence the extent of oxidative reactions. Previous studies have identified LAB, such as *Lactobacillus*, *Weissella*, and *Leuconostoc*, as core microbiota closely associated with sausage quality [5,6]. In recent years, the use of LAB strains with proven antioxidant capacity as starter cultures has emerged as a promising strategy to enhance both the quality and safety of fermented meat products. Numerous studies have demonstrated that many LAB possess intrinsic mechanisms for scavenging reactive oxygen species (ROS). They exert antioxidant effects through cell-surface properties and the secretion of active metabolites (such as exopolysaccharides and specific antioxidant enzymes), and the generation of antioxidant peptides, which neutralize free radicals or chelate pro-oxidant metal ions. Furthermore, they may indirectly mitigate lipid and protein oxidation by competitively inhibiting spoilage microorganisms, modulating pH, or altering pro-oxidant enzyme activity [7,8,9]. Among these, *Lactiplantibacillus plantarum* is one of the most extensively studied and widely applied LAB in fermented meat products, and its antioxidant properties have been well characterized [10]. However, relying solely on a few well-characterized meat-origin species may limit the discovery of novel antioxidative starters. Specifically, *Leuconostoc* spp. are known for their strong antioxidant capacities in various food systems; yet, their specific protective roles when applied to complex meat matrices remain comparatively underexplored.

In this study, we first compared in vitro antioxidative capacities of representative strains with potent activity from 5 LAB species (including three *Leuconostoc* species). Based on these results, we selected two strains, namely *Leuconostoc carnosum* AMS1 and *Lactiplantibacillus plantarum* P4 for subsequent sausage inoculation. We systematically evaluated their antioxidant efficacy during sausage fermentation and maturation, and their influence on microbial community succession. The findings are expected to broaden the application potential of functional LAB and to provide a technical reference for the application of natural antioxidant starter cultures to mitigate lipid and protein oxidation in fermented meat products.

## 2. Materials and Methods

### 2.1. Materials and Culture of Strains

Pork tender loin and back fat were obtained from 24-month-old local pigs in Yunnan. The bacterial strains were isolated from traditional fermented foods (Table 1). They were cryopreserved at −80 °C at the Food Microbial Culture Collection Center of Yunnan Academy of Agricultural Sciences.

### 2.2. Strain Cultivation

The strains were activated in MRS broth at 37 °C for three successive generations, with each generation cultured for 24 h.

### 2.3. Hydrogen Peroxide Tolerance Assay of LAB

MRS broth was autoclaved at 121 °C for 20 min before being supplemented with sterile H_2_O_2_ to obtain final concentrations of 0, 0.5, 1, and 2 mmol/L. The bacterial suspension was diluted to 1 × 10^8^ CFU/mL and then added to the H_2_O_2_-containing MRS broth at an inoculation ratio of 10% (*v*/*v*). After incubation at 37 °C for 8 h, bacterial growth was evaluated by determining the OD_600_ of the culture with a microplate reader.

### 2.4. Preparation of Cell-Free Supernatants (CFS), Intact Cells (IC), and Cell-Free Extracts (CFE) from Bacterial Strains

The bacterial strains were grown in MRS broth at 37 °C for 24 h and then harvested by centrifugation at 8000× *g* for 10 min at 4 °C. The resulting supernatant was carefully collected and designated as cell-free supernatant (CFS). The harvested cells were washed three times with PBS and subsequently resuspended in PBS to 1 × 10^8^ CFU/mL. The resulting cell suspension was separated into two fractions. One fraction was retained directly as intact cells (IC), whereas the other was disrupted by ultrasonication on ice. The disrupted cell suspension was centrifuged at 8000× *g* for 10 min at 4 °C, and the resulting supernatant was collected as the cell-free extract (CFE).

### 2.5. In Vitro Antioxidant Activity of Bacterial Preparations

#### 2.5.1. ABTS Radical Scavenging Activity Assay

The ABTS•+ radical solution was prepared by combining the ABTS solution with potassium persulfate, followed by incubation at room temperature in the dark for 16 h. Before the assay, the resulting solution was diluted with anhydrous ethanol until an absorbance of 0.70 ± 0.02 was reached at 734 nm. For the assay, 0.2 mL of the sample was added to 4 mL of the ABTS working solution and allowed to react for 30 min at room temperature in the dark. The absorbance was subsequently recorded, and the ABTS radical scavenging activity was determined using the equation below:

ABTS radical scavenging activity (%) =1−(A test/A control)×100

where A test = absorbance of the test sample; A control = absorbance of the control sample.

#### 2.5.2. DPPH Radical Scavenging Activity Assay

Briefly, 1 mL of the sample was combined with 2 mL of a DPPH solution prepared in ethanol at a concentration of 0.2 mmol/L. The mixture was kept in the dark at room temperature for 30 min and then centrifuged at 8000× *g* for 10 min at 4 °C. After centrifugation, the supernatant was collected, and its absorbance was determined at 517 nm, with deionized water used as the blank. The DPPH radical scavenging activity was subsequently calculated using the following equation:

DPPH scavenging activity (%) = 1−(A sample−A blank)/A control×100

where A sample = absorbance of the sample reaction; A blank = absorbance of the sample background control; A control = absorbance of the control reaction.

#### 2.5.3. Reducing Power Assay

A volume of 0.5 mL of the sample was combined with 0.5 mL of 0.2 M phosphate buffer (pH 6.6) and 0.5 mL of 1% potassium ferricyanide solution. The resulting mixture was maintained at 50 °C in a water bath for 20 min and subsequently allowed to cool to room temperature. Then, 0.5 mL of 10% trichloroacetic acid was added, and the mixture was centrifuged at 4000× *g* for 10 min. A 1 mL aliquot of the resulting supernatant was transferred to a fresh tube and mixed with 1 mL of distilled water and 1 mL of 0.1% ferric chloride solution. After standing for 10 min, the absorbance was determined at 700 nm.

### 2.6. Fermented Sausage Preparation

#### 2.6.1. Preparation of Cultures for Sausage Inoculation

To prepare the inocula of *L. carnosum* AMS1 and *L. plantarum* P4 for sausage fermentation, a 200 µL volume of each culture was inoculated into 10 mL of MRS broth and incubated at 28 °C for 48 h for AMS1, and at 37 °C for 24 h for P4. Each culture was then centrifuged at 6000 r/min for 10 min, and the supernatant was discarded. Subsequently, the pellet was washed three times with sterile saline and collected. Finally, each strain was resuspended in sterile saline to adjust the concentration to approximately 10^8^ CFU mL^−1^, as determined by plate counting on MRS agar.

#### 2.6.2. Sausage Formulation and Processing

Pork with a fat-to-lean ratio of 2:8 (*w*/*w*) was minced, and salt (2%, *w*/*w*) and glucose (1%, *w*/*w*) were added relative to the meat weight. The starter culture suspension was added at 3% (*w*/*w*) of the meat mixture, resulting in an initial inoculation level of approximately 6.0 × 10^7^ CFU/g of meat, as calculated from the predetermined cell density of the working suspension. For the control (CK) group, an equal mass (3%, *w*/*w*) of sterile saline was added instead of the starter culture suspension to maintain consistent moisture content and processing conditions across all treatments. No back-slopping or additional commercial starter cultures were used in any group.

All sausages were prepared on the same day using a single homogenized batch of standardized raw materials across three independent preparation rounds. The sausages were placed in a fermentation chamber for the controlled fermentation and early ripening stages. During the initial fermentation phase (first 24 h), the sausages were incubated at 30 °C with a relative humidity (RH) of 75–80%. Subsequently, during the early ripening phase (the remaining 6 days), the temperature was reduced to 15 °C with an RH of 60–65%. After this controlled fermentation and drying period, the sausages were further ripened at ambient temperature for 10 and 20 days, respectively. At each sampling interval (0, 3, 7, 17, and 27 days), one sausage—serving as the experimental unit—was randomly collected from each of the three preparation rounds, yielding three independent biological replicates (*n* = 3) per treatment group per time point. Because sampling was inherently destructive, distinct sausages were analyzed at each time point.

### 2.7. Bacterial DNA Extraction and High-Throughput Sequencing

Equal masses of homogenized sausage samples from the three independent biological replicates were pooled before extracting bacterial genomic DNA. Total genomic DNA was obtained with the TGuide S96 magnetic bead DNA extraction kit (Tiangen, Beijing, China) following the manufacturer’s instructions. The extracted DNA was examined for quality, purity, and concentration prior to amplification of the bacterial 16S rRNA gene. PCR was carried out with the universal primer pair 27F (5′-AGRGTTTGATYNTGGCTCAG-3′) and 1492R (5′-TASGGHTACCTTGTTASGACTT-3′). The amplification program consisted of an initial denaturation step at 95 °C for 2 min, followed by 30 cycles of denaturation at 98 °C for 10 s, annealing at 55 °C for 30 s, and extension at 72 °C for 90 s. A final extension was performed at 72 °C for 7 min.

The resulting amplicons were separated on a 1.8% agarose gel and purified using Agencourt AMPure XP beads. DNA concentrations were subsequently determined with the Qubit dsDNA HS Assay Kit (Invitrogen, Life Technologies Corporation, Eugene, OR, USA). Equal quantities of DNA amplicons from the samples were combined for library construction. SMRTbell libraries were prepared using the SMRTbell Express Template Prep Kit 2.0 and subsequently subjected to sequencing. Both library preparation and sequencing were conducted by Biomek Biotechnology Co., Ltd. (Beijing, China). Negative controls without DNA templates were included during DNA extraction and PCR amplification. No amplification products were detected in these negative controls.

Raw subreads generated by PacBio sequencing were processed using SMRT Link (version 8.0) to generate circular consensus sequencing (CCS) reads. The CCS reads were demultiplexed according to barcode sequences using lima (version 1.7.0), and primer sequences were removed. Quality filtering and chimera removal were subsequently performed to obtain high-quality full-length 16S rRNA sequences. Amplicon sequence variant (ASV) analysis was conducted using the DADA2 pipeline implemented in QIIME2 (version 2020.06), with ASVs occurring at less than 0.005% of total reads removed.

### 2.8. Measurement of Physicochemical Properties of Sausages

#### 2.8.1. pH, Water Activity and Moisture Content

The pH of sausages were determined using an electronic pH meter (Mettler Toledo, Columbus, OH, USA). Moisture content was determined using an electronic moisture meter (DHS-10A, Shanghai, China). Water activity (a_w_) analysis was performed using a water activity meter (GYM-1W, Shenzhen, China).

#### 2.8.2. Color Measurement

The color values of sausages were determined using a color reader (CM-5, Minolta, Kyoto, Japan). The sausage casing was removed, and the samples were cut into 1 cm-thick slices. Instrument calibration was performed using a white board. Color was characterized using the L*, a*, and b* parameters, which corresponded to lightness, redness/greenness, and yellowness/blueness, respectively. For each sample, three readings were taken at different surface locations and averaged to minimize intra-sample surface variation. The mean values from three independent sausages per treatment group were subjected to statistical analysis.

#### 2.8.3. Texture Profile Analysis

Texture profile analysis (TPA) was performed using a texture analyzer (TMS-Touch, FTC, Washington, DC, USA). After instrument calibration, sausage samples were subjected to a double-compression test using a 500 N load cell. The following parameters were set: probe dwell time of 10 s, test speed of 120 mm/min, compression ratio of 75%, interval time of 10 s, and trigger force of 0.75 N. For each sausage, three sub-samples were excised from different sections to perform technical replicate measurements. The readings from these three sub-samples were averaged to yield a single representative value for that individual sausage. Three independent sausages from separate preparation rounds were tested per treatment group at each time point.

### 2.9. Determination of Oxidation Indicators in Fermented Sausages

#### 2.9.1. Determination of Thiobarbituric Acid Reactive Substances (TBARS)

The TBARS content was analyzed based on a previously reported procedure with minor modifications [11]. Briefly, a 2 g portion of sausage was homogenized with 10 mL of 10% trichloroacetic acid (TCA) for 30 s. The resulting homogenate was centrifuged at 10,000× *g* for 5 min at 4 °C using a refrigerated centrifuge (HR/T20MM, Hexi, China). A 5 mL aliquot of the obtained supernatant was then combined with an equal volume of 0.02 mol/L thiobarbituric acid (TBA) solution and incubated in a boiling water bath for 20 min. Following heat treatment, the mixture was immediately cooled in an ice-water bath for 20 min, after which its absorbance was recorded at 532 nm. TBARS values were expressed as mg malondialdehyde (MDA) equivalents per kg of sample. For the blank, 5 mL of TCA solution was used in place of the sample supernatant.

#### 2.9.2. Determination of Lipoxygenase (LOX)

A 2 g portion of fermented sausage was homogenized with 15 mL of phosphate buffer (50 mM, pH 7.0) supplemented with 1 mM dithiothreitol (DTT) and 1 mM ethylenediaminetetraacetic acid (EDTA). The homogenate was maintained under stirring on ice for 30 min and subsequently centrifuged at 10,000× *g* for 60 min at 4 °C. After centrifugation, the supernatant was passed through glass fiber filters to obtain the crude enzyme extract, which was used for the determination of LOX activity. LOX activity was assayed following the procedure reported previously [12], with the oxidation of linoleic acid monitored by recording the increase in absorbance at 234 nm resulting from conjugated diene formation. The enzyme activity was determined from the variation in absorbance per minute and reported as U/g of sample.

#### 2.9.3. Determination of Carbonyl and Sulfhydryl Contents

Protein carbonyl content and sulfhydryl (SH) content were determined using commercial kits according to the manufacturers’ instructions (Solarbio, Beijing, China, catalog No. BC1270; Keming Biotechnology Co., Ltd., Suzhou, China, catalog No. TSH-1-G).

#### 2.9.4. Determination of Surface Hydrophobicity

Protein surface hydrophobicity was evaluated by the bromophenol blue (BPB) binding assay. The myofibrillar protein (MP) solution was first diluted to 1 mg/mL. Subsequently, 1 mL of the prepared MP solution was combined with 200 μL of BPB solution (1 mg/mL). A phosphate buffer solution containing no MP was prepared in parallel as the blank. The resulting mixtures were shaken at 25 °C for 10 min and then centrifuged at 6000 rpm for 15 min. The collected supernatants were diluted ten fold, and the absorbance was subsequently recorded at 595 nm. Protein surface hydrophobicity was determined from the quantity of BPB bound to MP according to the following equation:

$$B\;=\;200\times \frac{Ac-As}{Ac}$$

where B represents the amount of bromophenol blue bound (μg); Ac represents the absorbance of the blank control; and As represents the absorbance of the sample.

### 2.10. Statistical Analysis

All data are presented as mean ± standard deviation (SD) of three independent biological replicates (*n* = 3). For color and texture assays, three sub-sample measurements were averaged per sausage to account for spatial variation. For other physicochemical and oxidative assays, analytical determinations were performed in technical triplicates per homogenate or extract and averaged to yield a representative value for each individual sausage. Because different sausages were destructively sampled at each time point, data were evaluated under a balanced two-way factorial analysis of variance (ANOVA) framework, with data variance checked prior to Duncan’s post hoc test. Pairwise comparisons among treatment groups and time points were performed using Duncan’s multiple range test at a significance level of *p* < 0.05. Statistical analyses were conducted using R software (version 4.2.1), and ANOVA along with Duncan’s multiple range test was performed using the duncan.test function in the agricolae package (version 1.3–7). Beta diversity and overall bacterial community variations were evaluated via principal component analysis (PCA) based on the relative abundance of bacterial taxa using the BMKCloud platform (https://www.biocloud.net, accessed on 23 July 2026). Data visualization was performed using GraphPad Prism (version 8.0.2).

## 3. Results and Discussion

### 3.1. In Vitro Evaluation of Antioxidant Properties

#### 3.1.1. Hydrogen Peroxide Tolerance

Hydrogen peroxide (H_2_O_2_) is a common oxidant with high diffusibility and persistence. In the presence of metal ions, it can generate hydroxyl radicals, which cause severe oxidative damage to cells. Therefore, H_2_O_2_ tolerance is considered an important indicator for assessing the oxidative stress tolerance and antioxidant potential of LAB [13]. Table 2 shows the growth performance (OD_600_ values) of five LAB strains under different H_2_O_2_ concentrations (0–2 mM). Oxidative stress-induced growth inhibition was observed across all tested strains. The inhibitory effect on strain growth intensified with increasing H_2_O_2_ concentrations. At a concentration of 2 mM, the AMS1, L12, and P4 strains maintained OD_600_ values greater than 1.150, which was significantly higher than those of the other strains. This indicates that these strains exhibit robust tolerance to oxidative stress and may hold potential as functional starter cultures.

#### 3.1.2. Free Radical Scavenging Activity and Reducing Ability

ABTS and DPPH are widely used chemical models for evaluating free radical scavenging capacity. These radicals can undergo electron or hydrogen transfer reactions with antioxidant compounds, thereby reflecting the potential antioxidant activity of biological samples. LAB exhibit various antioxidant mechanisms, which help reduce the accumulation of free radicals during food fermentation and protect fermented food products from oxidative deterioration [14]. The reducing ability of enzymatic and non-enzymatic components toward ROS and metal ions is commonly considered an important indicator of antioxidant capacity [15].

The ABTS and DPPH radical scavenging activities and reducing capacities of different bacterial preparations are shown in Figure 1. For all tested strains, the antioxidant activities followed a consistent pattern, with CFSs exhibiting the highest activity, followed by ICs, whereas CFEs showed the lowest activity. Among the strains, AMS1 displayed the strongest antioxidant performance. Its CFS showed ABTS and DPPH radical scavenging rates of 90.38 ± 0.54%, 95.27 ± 0.13%, respectively, with a reducing power value of 0.73 ± 0.00. The CFS of strain P4 exhibited the second-highest activity, with radical scavenging rates exceeding 85% and a reducing capacity value above 0.6. These results suggest that the antioxidant activity of LAB strains was mainly associated with extracellular metabolites produced during growth. These metabolites may contribute to free radical scavenging and metal ion chelation, thereby suppressing oxidative reactions [14,16]. In comparison, the antioxidant contributions of ICs and CFEs were relatively minor under the present assay conditions. The radical scavenging activity of ICs may be associated with cell surface components, while their reducing power may be contributed by redox-active compounds located on the cell surface. The antioxidant activity of CFEs may originate from intracellular antioxidant systems, including reducing enzymes and low-molecular-weight antioxidants such as glutathione and NAD(P)H-related compounds [17]. Consistently, Wu et al. demonstrated that the reducing capacity was predominantly associated with CFSs, whereas only limited activity was detected in ICs and CFEs [18].

Overall, the in vitro antioxidant evaluations demonstrated that all five strains exhibited antioxidant potential. Among them, AMS1 and P4 showed superior performance in hydrogen peroxide tolerance, radical scavenging activity, and reducing ability. Considering that *L. carnosum* has been reported as a promising protective culture for meat products due to its antibacterial properties [19], AMS1 was selected for further evaluation of its antioxidant effects in sausages. The P4-inoculated group and naturally fermented group were included as comparative groups.

### 3.2. The Influence of Inoculation on the Bacterial Community During Sausage Fermentation

The influence of exogenously inoculated bacteria on the bacterial community structure during sausage fermentation was investigated using high-throughput sequencing technology. A total of 549,236 reads of bacterial 16S rRNA genes were obtained. After rigorous quality screening, 522,344 effective sequences were retained, accounting for 95.10% of the raw reads. The rarefaction curve (Figure 2A) reached a plateau, indicating that the sequencing depth was sufficient to characterize the bacterial diversity of the samples. As illustrated in the PCA score plot (Figure 2B), the bacterial communities from different treatment groups demonstrated distinct succession trajectories across the 7-day fermentation timeline.

The composition of bacterial communities at the phylum level is presented in Figure 2C. Across all samples, Firmicutes and Proteobacteria were the predominant bacterial phyla. Firmicutes constituted the dominant component in all treatment groups, although the temporal dynamics varied among the different groups. Notably, Proteobacteria was markedly enriched at day 0, and its relative abundance progressively declined with extended fermentation time. Similarly, Hu et al. reported that Firmicutes and Proteobacteria were also the dominant phyla in traditional dry sausages from Northeast China [20].

The bacterial community composition at the annotated species level is shown in Figure 2D. The top ten most abundant species were selected for analysis. In the CK group, *Macrococcus caseolyticus* dominated on day 0 (59.67%), but its relative abundance decreased to 21.77% by day 7, while *Lactococcus garvieae* rapidly proliferated to 64.9%, becoming the dominant species. Compared with the uninoculated control, inoculation with starter cultures influenced bacterial community succession during sausage fermentation. In the P4 group inoculated with *Lactiplantibacillus plantarum* P4, the relative abundance of this species steadily increased throughout fermentation, from 23.4% on day 0 to 27.15% on day 7, indicating its adaptability and persistence in the fermentation environment. *Lactiplantibacillus plantarum* exhibits extensive adaptability in various substrates, including vegetables, fruits, and meat products. Moreover, it can effectively tolerate dynamic changes in temperature, pH, and oxygen conditions during the fermentation process [21].

In the AMS1 group inoculated with *Leuconostoc carnosum* AMS1, this species accounted for 57.13% on day 0 and slightly decreased to 50.25% on day 3. Although its relative abundance dropped to 10.51% by day 7, it remained detectable throughout fermentation. The relative abundances of potential spoilage bacteria, such as *Macrococcus caseolyticus* and *Acinetobacter radioresistens*, were lower in the AMS1 group compared to the CK group. Aimondi et al. demonstrated that *Leuconostoc carnosum* possesses inhibitory effects against spoilage bacteria [22]. Although inoculation with AMS1 and P4 altered the microbial succession trajectory and reshaped the dominant bacterial community, *Staphylococcus saprophyticus* accounted for a substantial proportion of the bacterial community during the later stages of fermentation across all treatment groups. This phenomenon may be related to its ecological competitiveness in fermented sausages, which enables it to outcompete other microorganisms under conditions of reduced pH and low water activity [23]. Similar findings have also been reported in Harbin dry sausages and salami sausages [24].

### 3.3. The Influence of LAB Inoculation on the Physicochemical Properties During Sausage Fermentation and Maturation

#### 3.3.1. pH Changes

The physicochemical parameters of sausages significantly influence the quality of sausages. During fermentation, pH serves as a crucial indicator for monitoring the fermentation process. As shown in Figure 3, the pH values in all groups decreased rapidly during the first three days of fermentation, reaching their lowest levels on day 3. In both inoculated groups, the pH values dropped to as low as 4.72. From day 3 to day 17, pH values gradually increased. Throughout the fermentation period, the pH values of the inoculated groups were significantly lower than those of the uninoculated control group (*p* < 0.05). By day 17, significant differences in pH were observed among the three groups, with the AMS1-inoculated group exhibiting the lowest pH value at approximately 4.87. During the late maturation stage (days 17–27), pH values slightly increased across all groups.

The decline in pH during fermentation is mainly driven by organic acids produced via LAB growth and metabolism, which helps inhibit the proliferation of spoilage microorganisms and enhances product safety. The subsequent increase in pH during maturation may be associated with the generation of alkaline compounds, such as amines and biogenic amines, derived from protein degradation [25]. Xiao et al. reported that the pH of sausages inoculated with *Lactobacillus plantarum* and *Staphylococcus xylosus* decreased significantly during the early stage of fermentation, but subsequently increased to a certain extent in the later stage [26]. In summary, the inoculation of LAB significantly reduced pH values and maintained them at relatively low levels throughout subsequent stages, thereby contributing to the microbial safety of sausages during processing and storage.

#### 3.3.2. Moisture Content and Water Activity

Moisture content and water activity (a_w_) are key parameters affecting the fermentation, preservation, and sensory properties of fermented sausages. As shown in Figure 4, the moisture content of all groups decreased substantially within the first seven days. From day 7 to day 27, the moisture content in the AMS1 and P4 groups remained relatively stable, whereas that in the CK group exhibited a continuous decrease throughout both fermentation and maturation. Water activity gradually declined during the first 17 days, with a more pronounced reduction observed in the inoculated groups. Afterwards, a_w_ increased for all groups, and no significant differences were observed among the three groups at day 27.

The changes in moisture content and a_w_ during fermentation are likely related to LAB-mediated acidification and subsequent changes in muscle protein properties. The accumulation of organic acids reduced the pH toward the isoelectric point of MPs, decreasing electrostatic repulsion among protein molecules and promoting protein aggregation and contraction. These structural changes facilitated water expulsion from the protein matrix, contributing to the observed reductions in moisture content and a_w_ [27]. During the later stage, proteolytic degradation may partially disrupt the protein network and alter water distribution, which could account for the slight increase in a_w_. Overall, the inoculated groups exhibited faster moisture loss and more effective control of water activity, suggesting enhanced drying efficiency and favorable technological modifications in terms of water activity reduction. Xiao et al. also observed a significant decrease in a_w_ in sausages inoculated with *Lactobacillus plantarum* and *Staphylococcus xylosus* [26].

#### 3.3.3. Color Changes

Color is an important parameter for evaluating the quality of sausages. As shown in Figure 5, the L* value (lightness) decreased with prolonged fermentation time, and the inoculated groups consistently exhibited lower L* values than the CK group throughout the fermentation process. This change may be associated with accelerated drying, protein matrix modification, and changes in light scattering properties caused by fermentation and maturation [28]. The a* value (redness) showed an increasing trend, with a marked increase after day 7. Notably, the AMS1 group exhibited a significantly higher a* value than the other two groups during the later stage of maturation. The increased redness in the AMS1 group may result from a combination of reduced myoglobin oxidation and pH-dependent myoglobin redox stability [29]. The b* value (yellowness) increased slowly during fermentation in all groups. The b* value of the uninoculated group remained consistently higher than that of the inoculated groups. This difference may be associated with the antioxidant capacity of the starter cultures, which inhibited lipid oxidation and reduced the accumulation of oxidation-derived yellow pigments to some extent [30]. In general, a higher a* value and a lower b* value are generally considered desirable characteristics for fermented sausage color development. These results suggest that starter culture inoculation favorably influenced color development under the tested conditions. However, the sensory relevance of these color changes remains to be evaluated.

#### 3.3.4. Texture Changes

High-quality sausages are characterized by appropriate hardness, compact tissue structure, and elastic slices. As shown in Figure 6, the hardness of all groups increased significantly with prolonged maturation time, and significant differences in textural properties were observed among the groups. Throughout the entire processing period, the hardness of the inoculated groups was higher than that of the CK group, with the AMS1-inoculated group exhibiting the highest hardness. Regarding springiness, sausages fermented for three days showed the highest values; however, as fermentation progressed, springiness decreased by day 7. At day 3, the inoculated groups displayed higher springiness than the CK group. These results indicate that starter cultures played a role in sausage texture development. The acid-induced protein aggregation and contraction contributed to the formation of a denser protein network, resulting in increased hardness, improved firmness, and enhanced chewiness of the fermented sausages [26].

### 3.4. Inhibition of Lipid and Protein Oxidation in Sausages

#### 3.4.1. Lipid Oxidation Characteristics of Sausages

Lipid oxidation is one of the major factors affecting flavor development, rancidity formation, and quality deterioration in fermented sausages. To evaluate the antioxidant potential of the selected LAB in sausage fermentation, the effects of AMS1 and P4 on lipid oxidation were analyzed based on two indicators: thiobarbituric acid reactive substances (TBARS) value and lipoxygenase (LOX) activity.

The TBARS value reflects the content of malondialdehyde (MDA), a major secondary product of lipid oxidation, and is widely used as an indicator for evaluating lipid oxidation levels [31]. As shown in Figure 7A, the TBARS value gradually increased during 0–7 days and decreased later. Throughout the entire process, the TBARS values of the inoculated groups were significantly lower than those of the CK group. The temporary increase in TBARS during the early stage may be associated with the initial oxidation of unsaturated fatty acids and the formation of volatile flavor precursors [32]. The subsequent decline may result from the inhibition of oxidation reactions and the further transformation or degradation of secondary oxidation products during maturation.

LOX catalyzes the oxygenation of polyunsaturated fatty acids, leading to the formation of lipid hydroperoxides and subsequent oxidation products, which may contribute to off-flavor formation and quality deterioration in fermented meat products [33]. Figure 7B shows that LOX activity in sausage samples inoculated with AMS1 and P4 was significantly lower than that in the CK group throughout the processing (*p* < 0.05), and gradually declined over time. Notably, the AMS1 group exhibited the lowest LOX activity among all groups. This observed reduction may be partly driven by LAB-mediated acidification, which alters the optimal pH for LOX activity. Furthermore, antioxidant metabolites produced during fermentation may chelate pro-oxidant metal ions and scavenge free radicals, collectively limiting lipid oxidation [34]. This finding is consistent with Mei et al., who demonstrated that antioxidant LAB strains could effectively reduce LOX activity in fermented meat products [35]. However, the relative contributions of pH-dependent environmental changes versus strain-specific (e.g., antioxidant metabolite) effects cannot be fully distinguished in the present experimental design.

Previous studies have demonstrated that plant-associated *Lactiplantibacillus plantarum* can significantly inhibit lipid peroxidation in meat products, which may be related to its adaptation to oxidative stress conditions and its ability to produce antioxidant metabolites [36,37]. In conclusion, inoculation with AMS1 and P4 suppressed lipid oxidation during dry-cured sausage processing, thereby improving the oxidative stability of the products. Both strains showed potential as natural antioxidant starter cultures. Among them, the AMS1 group exhibited the strongest suppression of lipid oxidation parameters (TBARS and LOX activity), likely through a combination of pH-mediated environmental effects and strain-specific activities. Compared with the CK group, the TBARS value and LOX activity in the AMS1 group decreased by approximately 35% and 53%, respectively, at the end of processing, suggesting improved oxidative stability associated with AMS1 inoculation. To our knowledge, this is the first study to evaluate the in situ antioxidant potential of *L. carnosum* as a functional starter culture during sausage fermentation. While the in vitro antioxidant activity of the cell-free supernatant suggests the involvement of extracellular metabolites, the specific compounds responsible for this activity remain to be identified. The underlying mechanism may involve radical scavenging, pro-oxidant metal chelation, and microbial community modulation; however, further studies are required to fully elucidate these processes.

#### 3.4.2. Protein Oxidation Characteristics of Sausages

In fermented meat products, carbonyl content, sulfhydryl content, and surface hydrophobicity are used as key indicators for evaluating protein oxidation and conformational changes. When muscle proteins in sausages are exposed to ROS and secondary products generated from lipid oxidation, susceptible amino acid residues undergo oxidative modifications, resulting in the formation of carbonyl derivatives. Thus, an increase in carbonyl content is considered an indicator of enhanced protein oxidative damage [37]. As shown in Figure 8A, the carbonyl content gradually increased in all three sausage groups until day 17, when the highest values were observed, indicating that protein oxidation was most pronounced at this time. The subsequent decrease in carbonyl content observed on day 27 may be attributed to the further oxidation and transformation of carbonyl-containing proteins, as well as the formation of insoluble high-molecular-weight aggregates through protein–protein or protein–lipid interactions, which reduced the amount of detectable carbonyl groups.

Sulfhydryl groups are highly susceptible to oxidation and can be converted into disulfide bonds or other oxidized sulfur-containing compounds during processing. The sulfhydryl content decreased continuously in all groups; however, the reduction in the inoculated groups was less pronounced than that in the CK group. Notably, the AMS1 group maintained the highest sulfhydryl content across all stages (Figure 8B), suggesting that starter culture inoculation effectively alleviated oxidative modification of protein thiol groups.

Protein oxidation is often accompanied by conformational changes that expose previously buried hydrophobic groups, leading to an increased surface hydrophobicity [38]. In the present study, an increase in surface hydrophobicity was observed in all treatment groups, particularly during the late ripening stage (Figure 8C), indicating gradual unfolding and structural rearrangement of MPs. Nevertheless, the surface hydrophobicity of the inoculated groups remained consistently lower than that of the CK group, indicating that starter cultures mitigated oxidative structural alterations and helped preserve protein integrity.

Overall, all sausage groups exhibited varying degrees of protein oxidation, characterized by carbonyl accumulation, sulfhydryl loss, and increased surface hydrophobicity. This phenomenon may be associated with endogenous oxidative reactions and the propagation of lipid oxidation, both of which can generate ROS and reactive carbonyl compounds capable of inducing oxidative modifications of muscle proteins, particularly MPs. Notably, inoculation with starter cultures, especially AMS1, effectively inhibited protein oxidation, thereby contributing to the preservation of the structural integrity and functional properties of MPs [8].

### 3.5. Correlation Analysis Between Microbial Community and Antioxidant Performance

To explore potential associations between microbial community structure and oxidation indicators, RDA and Spearman correlation were conducted across the longitudinal sample matrix (*n* = 7, comprising the Day 0 uninoculated control alongside Days 3 and 7 for the CK, P4, and AMS1 groups) as exploratory tools to identify co-occurrence patterns between dominant bacterial taxa and key quality parameters. RDA (Figure 9A) revealed that carbonyl content and surface hydrophobicity vectors opposed sulfhydryl content, indicating that protein oxidation increased alongside sulfhydryl loss during fermentation. *Staphylococcus saprophyticus*, *Lactococcus garvieae*, *Lactiplantibacillus plantarum*, and *Leuconostoc carnosum* clustered with carbonyl content, surface hydrophobicity, and TBARS, while *Macrococcus caseolyticus* and *Acinetobacter radioresistens* aligned with LOX activity and sulfhydryl content, reflecting taxon-specific microenvironmental adaptations. However, because both microbial composition and oxidation indicators change progressively with fermentation time, these clustering patterns primarily reflect time-dependent co-variation rather than taxon-specific functional relationships.

Spearman analysis (Figure 9B) showed that *Acinetobacter radioresistens* correlated negatively with TBARS, carbonyl content, and surface hydrophobicity (*p* < 0.05), and positively with sulfhydryl content and LOX activity (*p* < 0.05). *Macrococcus caseolyticus* positively correlated with LOX activity (*p* < 0.01). *Staphylococcus kloosii* and *S. saprophyticus* exhibited positive correlations with TBARS, carbonyl content, and surface hydrophobicity, and negative correlations with sulfhydryl content and LOX activity, although these correlations did not reach statistical significance. Their persistence under low a_w_ and acidic conditions during the middle and late stages of fermentation may coincide with progressive lipid and protein oxidation.

The inoculated *L. plantarum* and *L. carnosum* correlated negatively with TBARS, carbonyl content, and surface hydrophobicity, and positively with sulfhydryl content, although these correlations were weak. During the early fermentation stage, oxidation levels were relatively low, masking the strains’ protective effects. As fermentation progressed, endogenous oxidation predominated over strain-specific regulation. Given the exploratory nature of this analysis and the restricted sample matrix, these weak correlations should not be overinterpreted as evidence of a direct in situ antioxidant effect of these strains. Rather, they may reflect indirect associations or coincidental co-variation with the progression of fermentation and maturation. Furthermore, oxidation indicators should be considered as dynamic markers rather than direct measurements of oxidation extent. LOX activity may be affected by continuous acidification, and its decline during fermentation does not necessarily indicate inhibition of lipid oxidation. Similarly, TBARS may reflect the accumulation of lipid oxidation intermediates and not fully represent the overall lipid oxidation status. Notably, all microbiome-related analyses were based on a single composite sample per timepoint per treatment group. Consequently, the observed associations between bacterial taxa and physicochemical/oxidation parameters are descriptive and hypothesis-generating rather than confirmatory. These preliminary observations nevertheless provide a conceptual framework to guide subsequent research incorporating multi-replicate validation to establish causal relationships.

## 4. Conclusions

This study first analyzed the distribution of in vitro antioxidant activities among different cell fractions of five antioxidative LAB strains and revealed that these activities were mainly associated with their extracellular metabolites. Subsequently, two LAB strains, AMS1 and P4, which showed good in vitro antioxidant activity and oxidative tolerance, were selected as sole starter cultures to evaluate their effects on antioxidant performance in sausages.

The results showed that inoculation with AMS1 and P4 influenced the fermentation process, as evidenced by lower pH and altered color parameters (increased redness and decreased yellowness) and texture (increased hardness and springiness). The inoculated groups also exhibited faster moisture loss and more effective water activity reduction under the conditions tested, suggesting favorable technological modifications in drying characteristics. High-throughput sequencing revealed that the strains adapted well to the sausage fermentation environment and influenced bacterial community succession, with lower relative abundance of certain spoilage-related taxa observed in the AMS1 group compared to the CK group. However, this observation does not necessarily indicate a reduction in absolute cell numbers.

Regarding lipid oxidation, the AMS1 and P4 groups showed lower TBARS values and LOX activity than the CK group. Among them, the AMS1 group exhibited the most pronounced reduction on day 27, with TBARS and LOX activity decreased by approximately 35% and 53%, respectively, compared with the uninoculated group. These changes may be related to the antioxidant capacity of the strain, together with the reduced stability of LOX under continued acidification. Regarding protein oxidation, inoculation was associated with lower carbonyl accumulation, less sulfhydryl loss, and a smaller increase in surface hydrophobicity, suggesting an alleviation of protein oxidative modification. In conclusion, AMS1 and P4, particularly AMS1, showed potential to improve oxidative stability in dry-cured sausages by modulating the fermentation microenvironment and attenuating lipid and protein oxidation during fermentation and maturation, supporting their potential application as antioxidant starter cultures.

While the observed physicochemical modifications and oxidative delays offer promising technological insights, the microbiome-related findings from this study should be interpreted as exploratory and hypothesis-generating. To build upon these preliminary observations, future research should incorporate expanded independent biological batches, particularly under conventional nitrite-containing conditions, to validate these trends. Further studies are also needed to evaluate the effects of these specific strains on the overall organoleptic quality of the product via sensory evaluation. Furthermore, in-depth studies are warranted to elucidate the underlying antioxidant mechanisms, including the characterization of antioxidant metabolites produced by the strains and their roles in regulating lipid and protein oxidation during sausage fermentation and maturation.

## Figures and Tables

**Figure 1:**
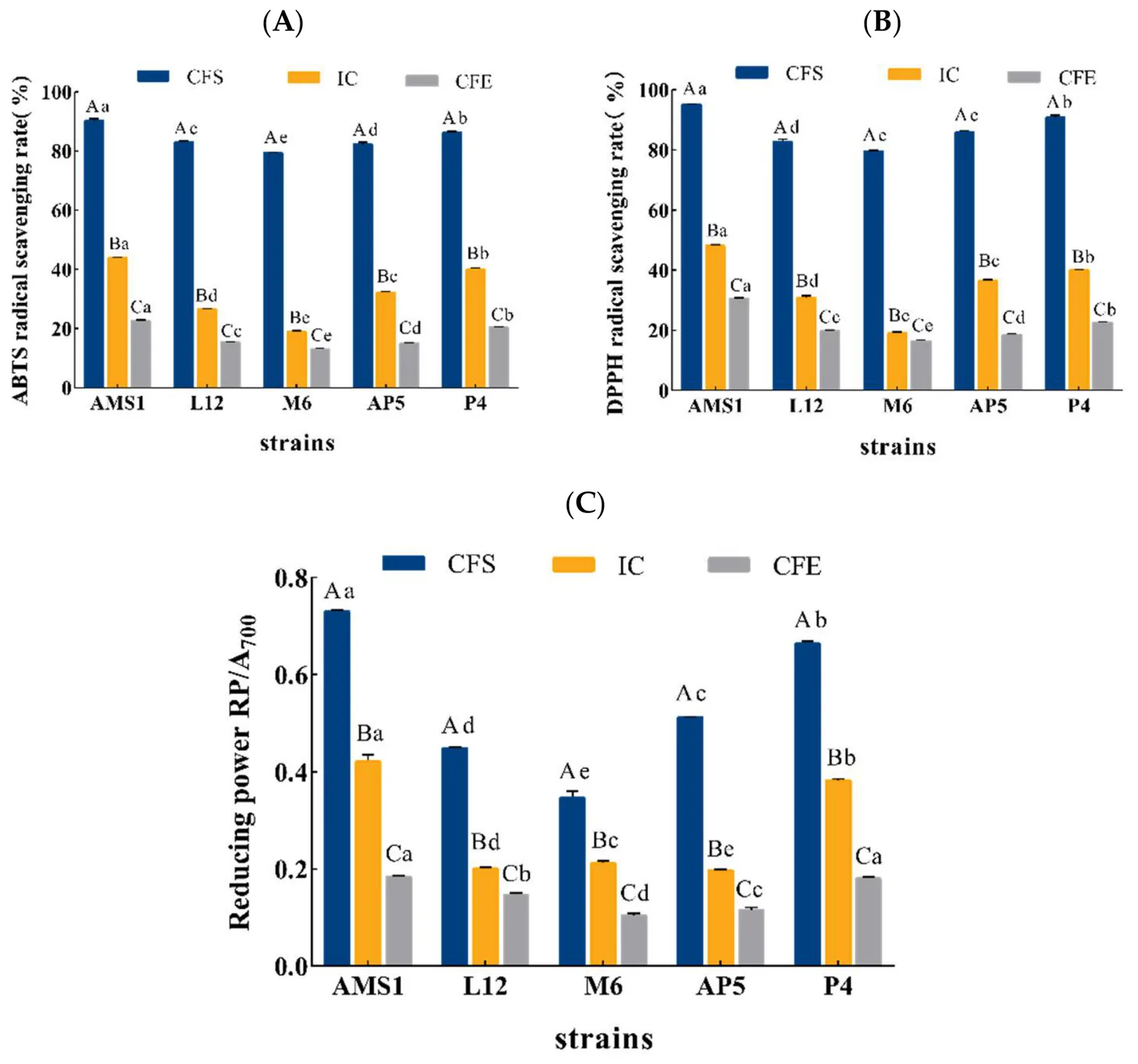
In vitro antioxidant activities of LAB strains: (**A**) ABTS radical scavenging activity, (**B**) DPPH radical scavenging activity, and (**C**) reducing power. Different capital letters represent significant differences among preparations of the same strain, while different lowercase letters represent significant differences among strains.

**Figure 2:**
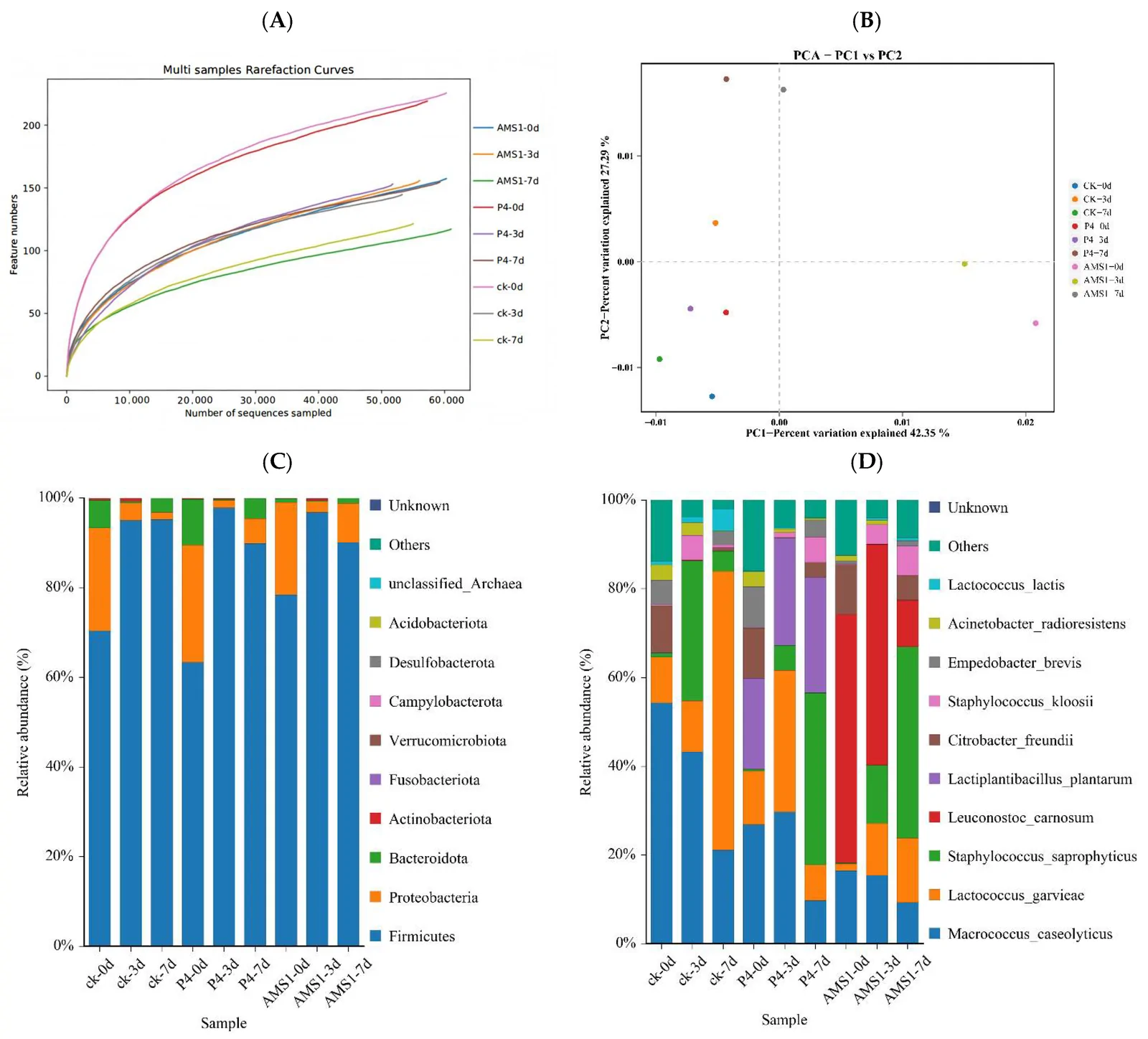
Bacterial community analysis during sausage fermentation and early ripening: (**A**) rarefaction curves, (**B**) bacterial β-diversity, (**C**) relative abundance of bacterial communities at the phylum level, and (**D**) relative abundance at the species level.

**Figure 3:**
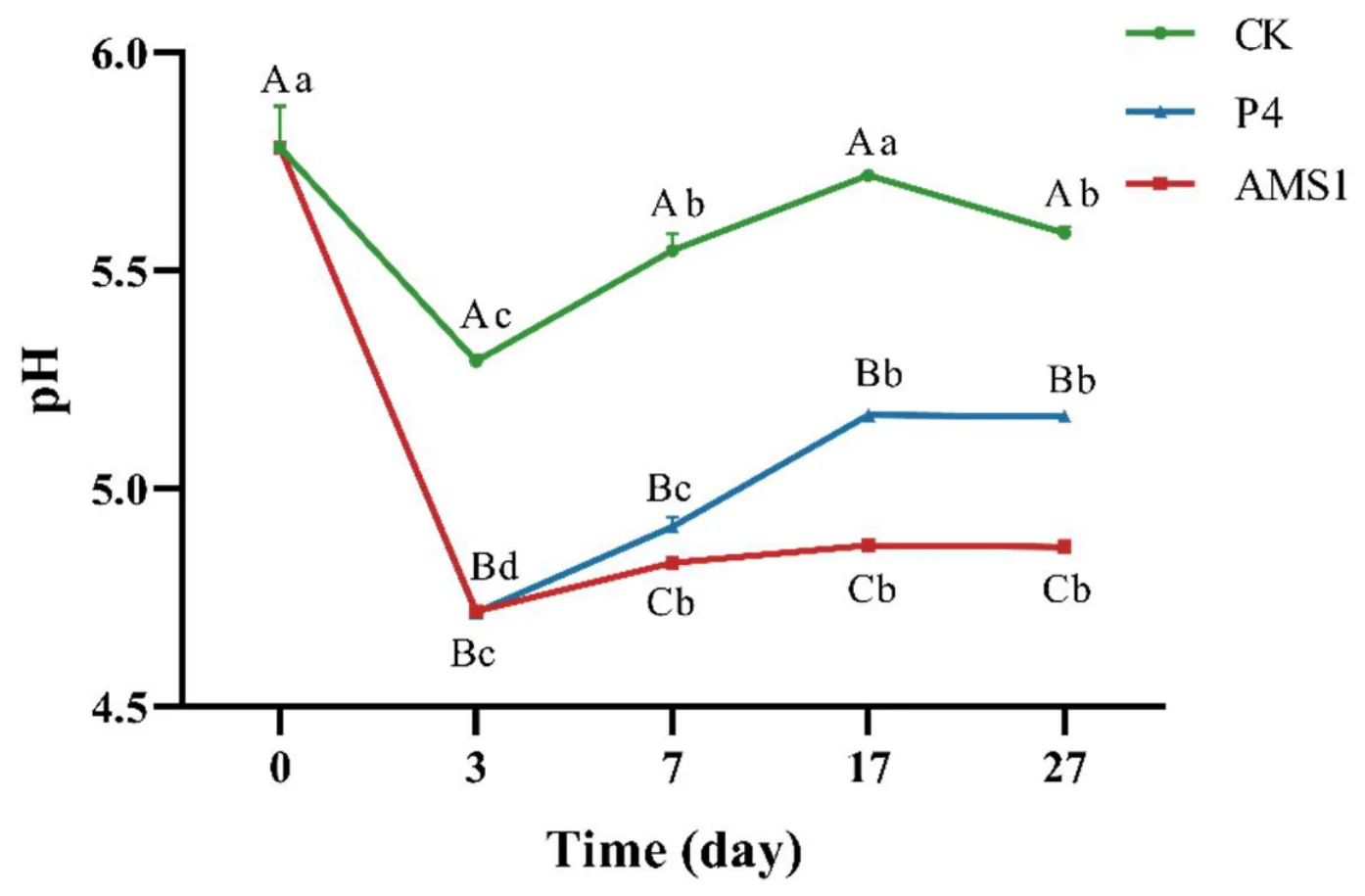
pH values during sausage fermentation and maturation. Different capital letters indicate significant differences among strains, while different lowercase letters indicate significant differences among different time points. The same notation applies hereafter.

**Figure 4:**
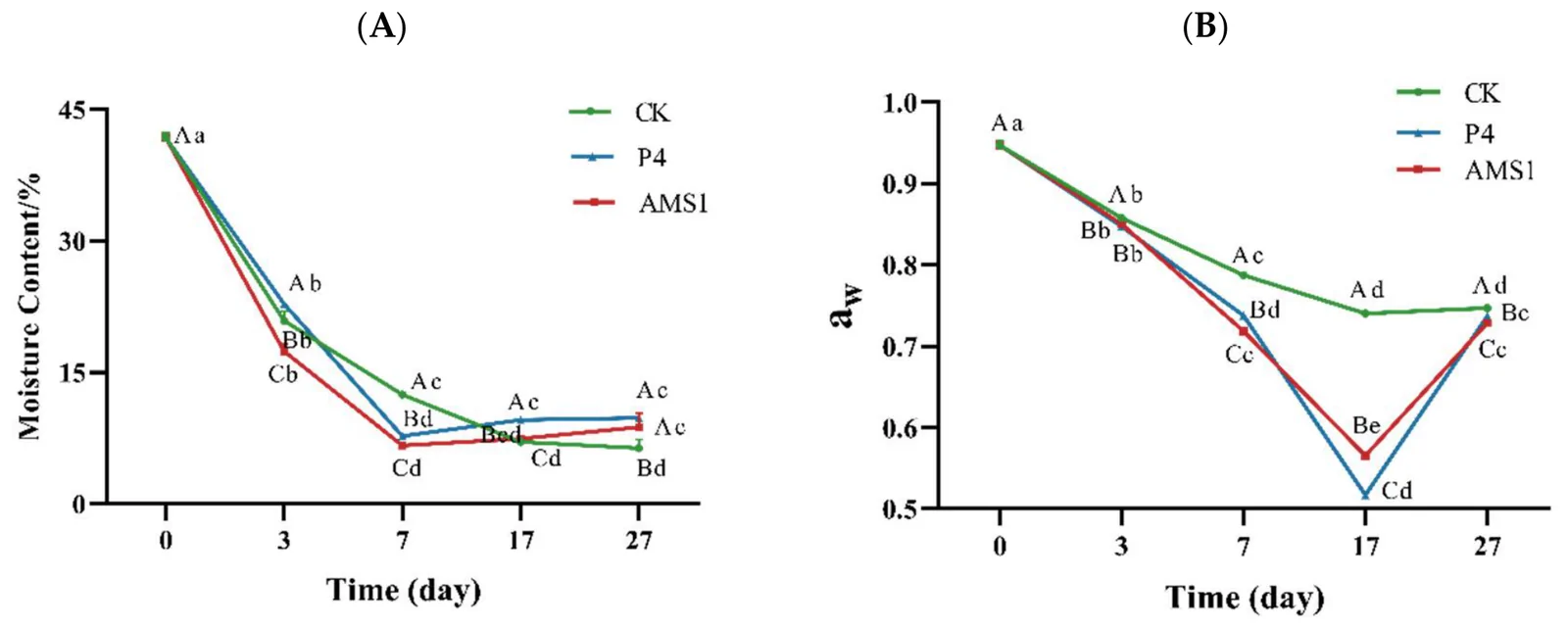
Changes in (**A**) moisture content and (**B**) water activity of sausages.

**Figure 5:**
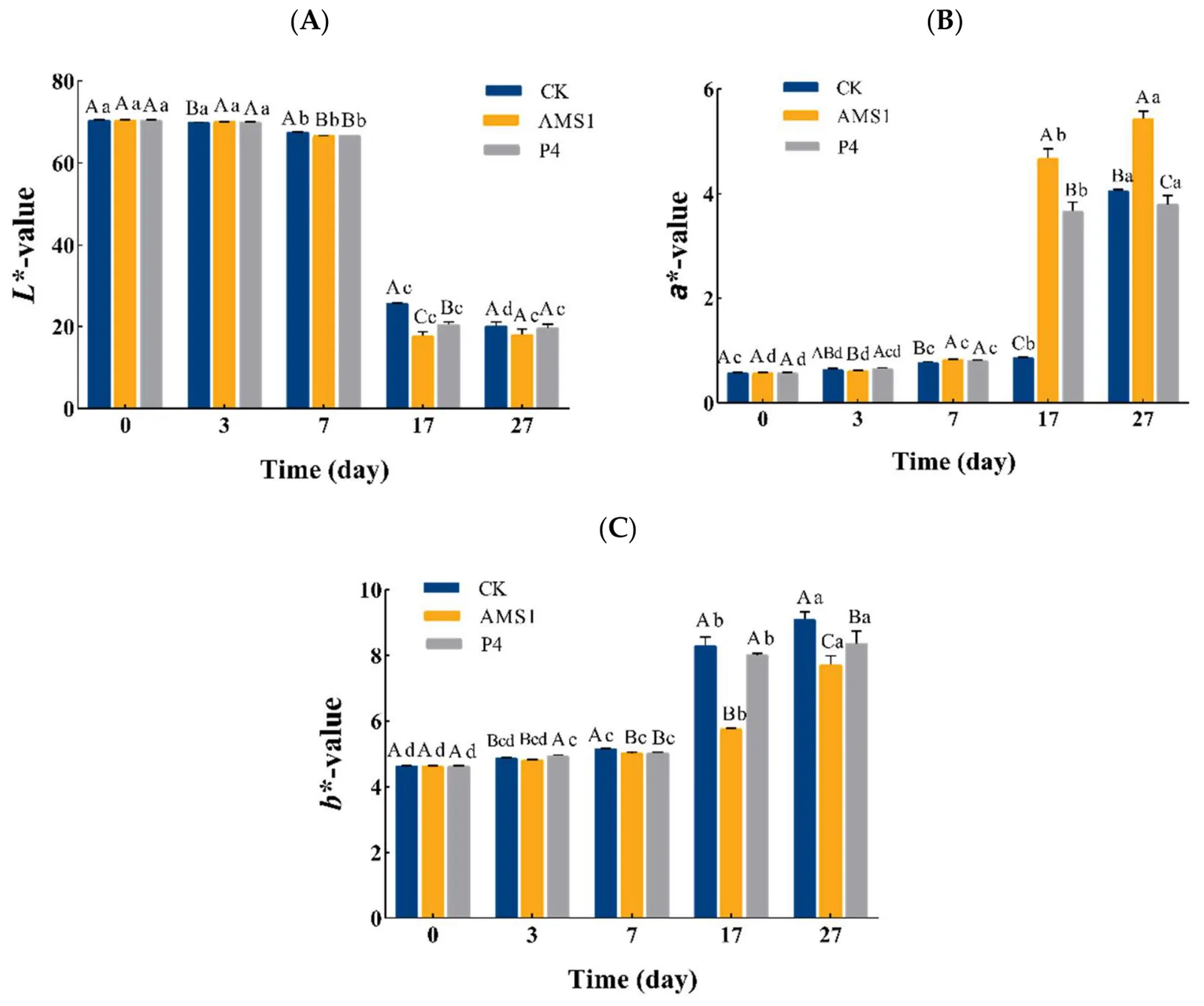
Changes in (**A**) L* values, (**B**) a* values, and (**C**) b* values of sausages.

**Figure 6:**
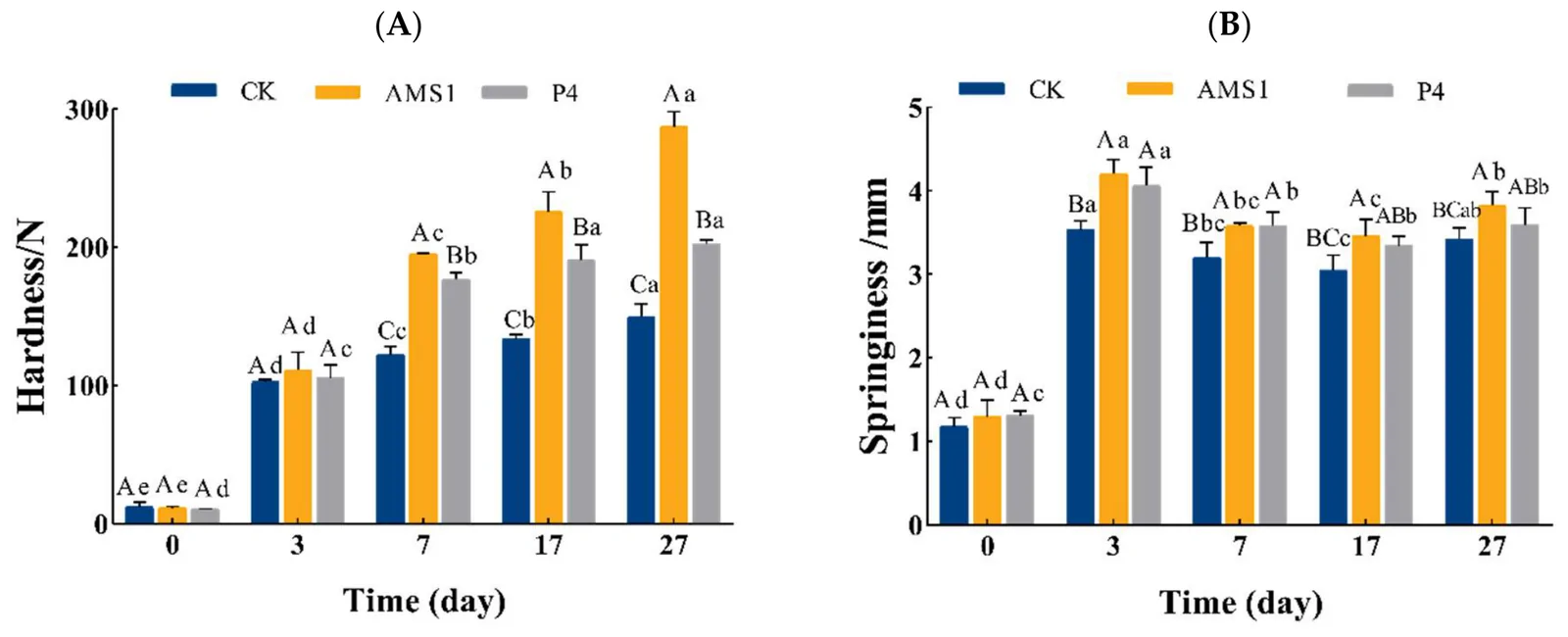
Changes in (**A**) hardness and (**B**) springiness of sausages.

**Figure 7:**
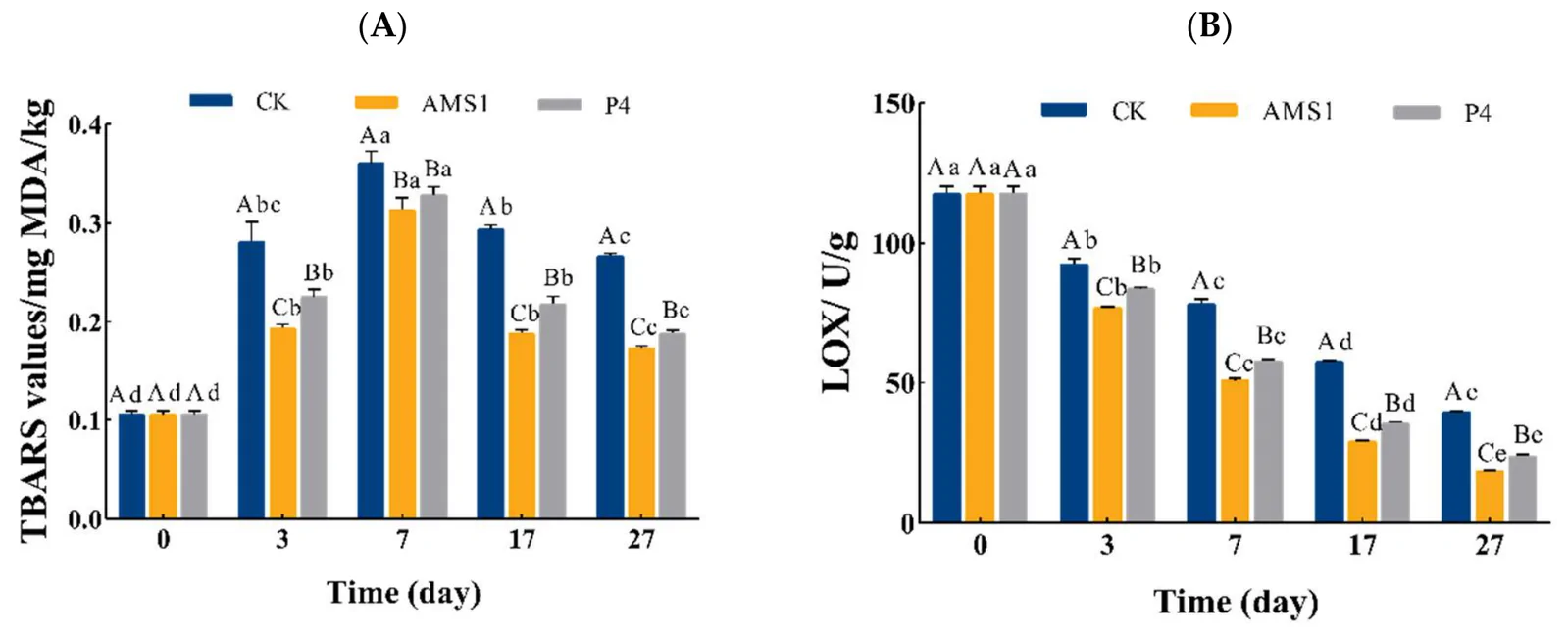
Changes in (**A**) thiobarbituric acid reactive substances (TBARS) and (**B**) lipoxygenase (LOX) during sausage fermentation and maturation.

**Figure 8:**
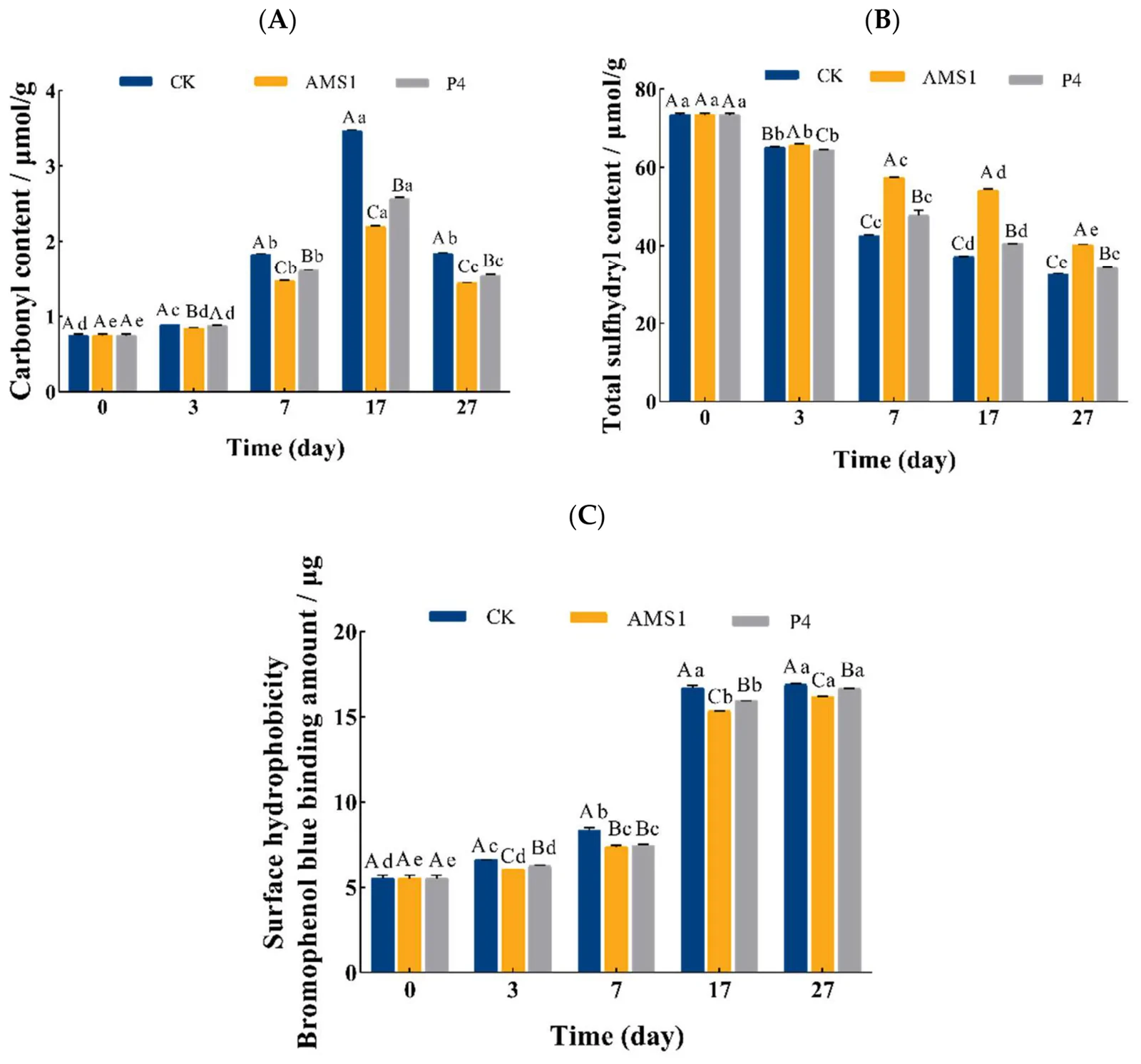
Changes in protein oxidation markers of sausages: (**A**) carbonyl content, (**B**) total sulfhydryl content, and (**C**) surface hydrophobicity of sausages.

**Figure 9:**
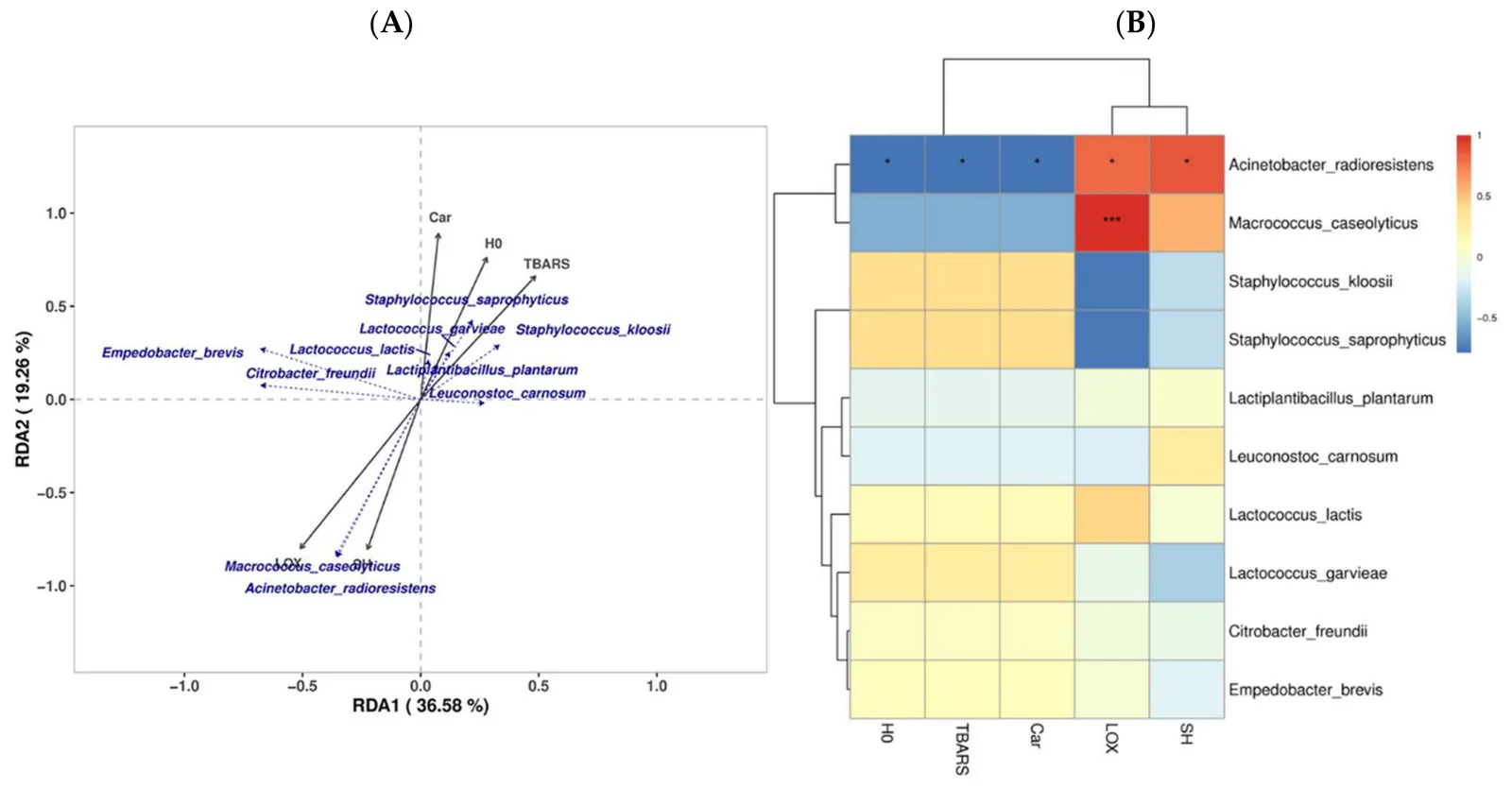
Analysis of the relationship between oxidation indicators and the top 10 most abundant bacterial species: (**A**) Redundancy analysis (RDA) and (**B**) Spearman correlation analysis. Note: Car, carbonyl content; SH, total sulfhydryl content; H0, surface hydrophobicity index. Asterisks denote statistically significant Spearman rank correlations (* *p* < 0.05, *** *p* < 0.001).

**Table 1:** LAB strains tested in this study.

Abbreviation	Strain Number	Isolation Source
P4	*Lactiplantibacillus plantarum* P4	Pickled vegetable
M6	*Latilactobacillus sakei* M6	Fermented rice
AMS1	*Leuconostoc carnosum* AMS1	Fermented sausage
AP5	*Leuconostoc mesenteroides* AP5	Pickled peach
L12	*Leuconostoc citreum* L12	Pickled peach

**Table 2:** OD_600_ Values of LAB at Different H_2_O_2_ Concentrations.

Strain	0 mM	0.5 mM	1 mM	2 mM
AMS1	1.412 ± 0.002 ^Aa^	1.344 ± 0.001 ^Ab^	1.271 ± 0.001 ^Ac^	1.209 ± 0.002 ^Ad^
L12	1.411 ± 0.002 ^Aa^	1.306 ± 0.002 ^Cb^	1.219 ± 0.002 ^Dc^	1.153 ± 0.003 ^Cd^
M6	1.401 ± 0.002 ^Aa^	1.277 ± 0.002 ^Db^	1.194 ± 0.002 ^Ec^	1.033 ± 0.002 ^Ed^
AP5	1.407 ± 0.002 ^Aa^	1.301 ± 0.001 ^Bb^	1.236 ± 0.003 ^Bc^	1.115 ± 0.001 ^Dd^
P4	1.407 ± 0.012 ^Aa^	1.299 ± 0.003 ^Bb^	1.227 ± 0.002 ^Cc^	1.172 ± 0.002 ^Bd^

Note: Different uppercase letters (A–E) represent significant differences among strains, while different lowercase letters (a–d) represent significant differences among concentrations.

## Data Availability

The original data obtained in the study are included in the article. Further inquiries can be directed to the corresponding authors.

## Abbreviations

The following abbreviations are used in this manuscript:

LAB	Lactic acid bacteria
TBARS	Thiobarbituric Acid Reactive Substances
LOX	Lipoxygenase
ROS	Reactive oxygen species
RP	Reducing Power
SH	Sulfhydryl
Car	Carbonyl content
H0	Surface Hydrophobicity
